# Sentiment Analysis: Radiological Narratives And Intracranial MRI Hemorrhage Images for Dementia Alzheimer’S Prevention

**DOI:** 10.2174/0115734056420799250902080814

**Published:** 2025-10-15

**Authors:** K. Balasaranya, P. Ezhumalai, N.R. Shanker

**Affiliations:** 1 Department of CSE, R.M.D. Engineering College, Kavareipattai, Chennai, India; 2 Department of CSE, Aalim Muhammed Salegh College of Engineering, Chennai, India; £ Present Address: Department of CSE, Sriram Engineering College, Perumalpattu, Veppampattu [R.S], Chennai, India

**Keywords:** Intracranial hemorrhage, Prognosis, Therapeutic sentiment, Dementia prediction, Vision Image Transformer (ViT), Clinical narratives

## Abstract

**Introduction:**

Intracranial hemorrhage (IH) causes dementia and Alzheimer’s disease in the later stages. Until now, the accurate, early detection of IH, its prognosis, and therapeutic interventions have been a challenging task. Objective: A Multimodal Joint Fusion Sentiment Analysis (MJFSA) framework is proposed for the early detection and classification of IH, as well as sentiment analysis to support prognosis and therapeutic report generation.

**Methodology:**

MJFSA integrates radiological images and the radiological clinical narrative reports (RCNRs). In the proposed MJFSA model, MRI brain images are enhanced using the modified Contrast Limited Adaptive Histogram Equalization (M-CLAHE) algorithm. Enhanced images are processed with the proposed Tuned Temporal-GAN (Tuned-T-GAN) algorithm to generate temporal images. RCNRs are generated for temporal images using the Microsoft-Phi2 language model. Temporal images are processed with the Tuned-Vision Image Transformer (T-ViT) model to extract image features. On the other hand, the Bio-Bidirectional Encoder Representation Transformer (Bio-BERT) processes the RCNR texts for text feature extraction. Temporal image and RCNR text features are used for IH classification, such as intracerebral hemorrhage (ICH), epidural hemorrhage (EDH), subdural hemorrhage (SDH), and intraventricular hemorrhage (IVH), resulting in sentiment analysis for prognosis and therapeutic reports.

**Results:**

The MJFSA model has achieved an accuracy of 96.5% in prognosis sentiment analysis and 94.5% in therapeutic sentiment analysis.

**Discussion:**

The Multimodal Joint Fusion Sentiment Analysis (MJFSA) framework detects IH and classifies it using sentiment analysis for prognosis and therapeutic report generation.

**Conclusion:**

The MJFSA model’s prognosis and therapeutic sentiment analysis report aims to support the early identification and management of risk factors associated with dementia and Alzheimer’s disease.

## 1 INTRODUCTION

Intracranial hemorrhage (IH) is a serious condition characterized by bleeding in the human brain and surrounding areas, thereby leading to stroke. IH is divided into four types: intracerebral hemorrhage (ICH), epidural hemorrhage (EDH), subdural hemorrhage (SDH), and intraventricular hemorrhage (IVH) [1]. ICH occurs when there is an accumulation of blood within the brain parenchyma, which often results in a stroke. In EDH, bleeding occurs between the inner skull and the outer layer of the dura mater. SDH results from blood accumulation in the subdural space of the brain. The presence of blood in the intraventricular septum is known as intraventricular hemorrhage (IVH). IH increases the risk of death by 50% due to hematoma expansion within 72 hours [2]. Brain cells begin to die within 3-4 minutes due to reduced oxygen supply [3]. Dementia develops approximately 5.6 years after the patient’s intracranial hemorrhage [4]. To improve the survival rate of patients, artificial intelligence-based CT/MRI analysis reduces diagnostic delays and enhances diagnostic accuracy. Table **1** presents the types of hemorrhage and their risk of leading to dementia or Alzheimer’s disease.

Physicians diagnose intracranial hemorrhage (IH) at an earlier stage using MR/CT images and prevent dementia and Alzheimer’s diseases through clinical assessments and neurological examinations. The radiological clinical narrative reports (RCNRs) provide visual information regarding intracranial hemorrhage, including interpretations, recommendations, and impressions [5]. Visual interpretation of IH can lead to an inaccurate diagnosis due to similar features of blood clots and calcification in brain images [6]. IH and dementia are analyzed through RCNRs.

Traditional radiology report findings have limitations, including difficulties in interpreting small changes in early cases of low-contrast CT images and ambiguity in the language used to describe hemorrhage type and size in relation to clinical relevance. The integration of other clinical reports, such as progression notes, consultation notes, and procedural notes, requires more time for physicians to analyze and interpret. Delays in updating documentation lead to missed and delayed diagnoses of IH [7]. To improve the diagnostic accuracy of IH, prevent dementia and Alzheimer’s disease over time, and reduce human error, images and reports are used in combination for the diagnosis of IH. Early diagnosis of IH is essential for the prevention of dementia and Alzheimer’s disease over time [8].

Medical sentiment analysis is categorized into (i) patient-specific sentiment analysis, (ii) clinical sentiment analysis, (iii) contextual sentiment analysis, and (iv) casual sentiment analysis [9]. Clinical sentiment analysis in healthcare involves examining the textual content of clinical narratives, such as discharge summaries, radiology reports, and nurse notes. These narratives include information about changes in health status, treatment effectiveness, and the presence and prognosis of disease. Clinical narrative-based sentiments differ from traditional sentiment analysis because words have varying meanings depending on a patient's medical history and domain-specific linguistic nuances. Therefore, sentiment from clinical narratives is identified through the use of unified medical language systems (UMLS). In the medical context, sentiment can be either implicit or explicit. Clinical sentiment analysis is further divided into three types: (i) prognosis sentiment analysis, (ii) therapeutic sentiment analysis, and (iii) disease progression sentiment analysis [10].

Prognosis sentiment analysis refers to the automatic evaluation of a patient's health condition as expressed in clinical text. It categorizes sentiments as positive, negative, uncertain, or neutral based on physicians' opinions regarding the patient's status, such as improving, worsening, or stable. Prognosis-related sentiments are extracted using various techniques, including domain-specific sentiment analysis, aspect-based sentiment analysis, negation and modality handling, and context-aware models [11]. Domain-specific IH lexicons include positive terms, such as “good outcome,” “favorable outcome,” “improved survival,” “recovery,” and “stable.” Negative IH lexicons consist of domain-related words like “coma,” “large hematoma,” “intraventricular hemorrhage,” “posterior fossa hemorrhage,” and “old age.” Neutral IH lexicons encompass terms, such as “Glasgow Coma Scale (GCS),” “ICH score,” “hematoma volume,” “infratentorial/supratentorial,” “midline shift,” “mass effect,” and “perihematomal edema” [12]. Prognosis analyzes the level of consciousness, size, and location, such as ventricles and lobar locations of the brain [13].

Therapeutic sentiment analysis involves evaluating patient well-being during treatments and aims to enhance the health of patients [14]. Sentiment is classified based on the effectiveness and side effects of these treatments. Positive therapeutic sentiments include improvement, care, and successful management of treatment complications. In contrast, negative therapeutic sentiments encompass defects, complications, and poor diagnoses [15]. Positive therapeutic sentiment enhances patients' quality of life and leads to better psychological outcomes, while negative therapeutic sentiment increases depression and anxiety, reducing the quality of life for behavioral health patients.

Clinical analysis in IH involves informed decisions regarding patient health conditions in complex situations. Clinical decision-making for intracerebral hemorrhage has several steps, such as assessment, neuroimaging, risk identification, and acute management. AI-based deep learning imaging algorithms are used to detect IH conditions in emergencies and during surgery to identify any missing lesions [16]. Recent techniques in clinical decision-making enhance the identification of prognosis and therapies for IH, utilizing susceptibility-weighted imaging for microbleeds and diffusion-weighted imaging to differentiate between acute and primary intracerebral hemorrhage. FLAIR imaging provides a detailed visualization of IH. The aforementioned advanced imaging techniques present several challenges, including variable sensitivity and specificity due to the stage, size, and location of the hemorrhage. Inaccurate diagnoses occur due to signal variability in imaging, missed lesions, and anatomical overlaps caused by calcifications. AI imaging methods increase the number of false positives and negatives, which, in turn, increases the workload for radiologists for accurate clinical decisions. Therefore, clinical narratives need to be included to provide context for IH radiology images and to improve diagnostic accuracy, preventing dementia and Alzheimer’s disease over time. To understand the relationship between imaging and clinical narratives, clinical sentiment analysis is needed, such as prognosis sentiment analysis and therapeutic sentiment analysis reports.

### 1.1 Research Questions

1. How can the clinical narrative in radiology reports of image datasets provided by the radiologist be improved for prognosis and therapeutic sentiment analysis of intracranial hemorrhage based on LLM and DL algorithms to prevent dementia and Alzheimer’s disease over time?
2. How does the integration of LLM and DL provide additional information from other clinical narrative reports of intracranial hemorrhage patients for effective prediction of prognosis and therapeutic sentiment analysis of a patient when writing the impressions of medical reports to prevent dementia and Alzheimer’s disease over time?
3. What models can efficiently integrate the text data features correspondingly to the image dataset features for effective intracranial hemorrhage detection at an early stage and prevent dementia and Alzheimer’s disease over time?

### 1.2 Problem Statement

Although AI imaging techniques enhance intracranial hemorrhage (IH) images for accurate detection, they still face challenges, such as identifying subtypes of hemorrhage patterns [18], data quality [19], and the availability of annotations [20]. These challenges reduce the diagnostic accuracy of intracranial hemorrhage, leading to an increase in false positives and false negatives, ultimately delaying accurate IH detection [17]. The classification of intracranial hemorrhage subtypes incorporates multimodal data, such as radiology images, impression reports, and other documents with sentiment scores, to classify the subtypes of intracerebral hemorrhage (ICH), epidural hemorrhage (EDH), subdural hemorrhage (SDH), and intraventricular hemorrhage (IVH), and to prevent dementia and Alzheimer’s disease over time.

### 1.3 Contributions

In this study, text features from RCNRs, progression notes, consultation notes, and procedural notes are combined with imaging features to generate sentiment-based prognosis and therapeutic reports. The progression note includes updates on symptoms, neurological findings, imaging results, and assessments of progression [21]. The consultation note encompasses the patient's history, physical examination, assessment, and plan. The procedural note clearly indicates the size of the hematoma, describes the surgical approach, details intraoperative findings, and outlines postoperative monitoring and follow-up plans.

1. To improve radiology clinical narrative reports for prognosis and therapeutic sentiment analysis, large language models (LLM) and deep learning (DL) are combined to predict dementia and Alzheimer’s disease over time, and also detect types of intracranial hemorrhage. The proposed tuned temporal generative adversarial network (Tuned T-GAN) algorithm generates images to predict dementia by leveraging temporal information. Clinical notes, including consultation notes, progression notes, and procedural notes, are generated using the Microsoft/Phi-2 model.
2. To effectively predict the progression of sentiment analysis, therapeutic reports, and dementia and Alzheimer’s disease over time, the BioBERT model is proposed to extract disease progression from progression notes, identify patterns in radiology reports, and determine treatment from procedure and consultation notes. The proposed Tuned-Vision Image Transformer (T-ViT) method extracts structural feature information, such as hemorrhage size and location.
3. To effectively predict small and early-stage intracranial hemorrhage and predict dementia and Alzheimer’s disease over time, the ViT and Bio-BERT models align images with text features and descriptions of intracranial hemorrhage (IH). RCNR text and image features are processed through a multimodal classifier that classifies prognosis and therapeutic sentiments based on the similarity scores of combined text and image features, thereby predicting dementia and Alzheimer’s disease. Finally, the proposed MJFSA generates a new report with prognosis, therapeutic sentiment, and risk of hemorrhage, dementia, and Alzheimer’s disease.

The paper is organized as follows: Section 2 describes the state-of-the-art techniques for the prevention of intracranial hemorrhage and dementia, as well as Alzheimer’s disease, through prognostic and therapeutic treatments. Section 3 outlines the methodology for prognosis and therapeutic sentiment analysis of radiological clinical narrative reports related to intracranial hemorrhage, aimed at predicting dementia and Alzheimer’s disease over time. Section 4 presents the results and discussion regarding the classification of intracranial hemorrhage and the prediction of dementia and Alzheimer’s disease over time. Finally, Section 5 provides the conclusion.

## 2 METHODOLOGY

Flow diagram of the proposed prognosis and therapeutic sentiment analysis reports of intracranial hemorrhage to predict dementia and Alzheimer’s disease over time is shown in Fig. (**1a** and **b**). The Radiological Intracranial Hemorrhage Impression Report (RIHIR) contains brain MRI images accompanied by text. The RIHIR includes information about type, location, size, and findings, such as compression of ventricles, herniation, and skull fracture. The appearance of intracranial hemorrhage images varies depending on the age of the hemorrhage, the type of imaging modality, changes in hemoglobin state, and clot formation. For the early and accurate detection of intracranial hemorrhage, a Contrast-Limited Adaptive Histogram Equalization (CLAHE) algorithm is used to enhance small-sized hemorrhages by improving local contrast without adding noise.

After image enhancement, temporal intracranial hemorrhage images are generated using the proposed Tuned-T-GAN algorithm and analyzed to examine the temporal and spatial information of intracranial hemorrhage over time, as well as prognosis pre- and post-dementia and Alzheimer’s diseases resulting from vascular damage. Hence, the temporal IH Tuned T-GAN-based images are used to prevent dementia and Alzheimer’s diseases at an earlier stage through imaging-based prognosis. Additionally, the Microsoft Phi2 model is used to generate clinical notes, such as consultation, progression, and procedural notes, for the generated temporal images. The temporal images and generated clinical notes are applied using the Tuned-ViT and BioBERT transformer models for feature extraction, improving the accuracy of disease prognosis and therapeutic treatment recommendations. Finally, the prognosis and therapeutic sentiment analysis reports are generated based on the words present in the text and their associated scores, which help predict the likelihood of dementia and Alzheimer’s diseases.

### 2.1 Dataset Description

The Radiology Object in Context Version-2 (ROCO-v2) dataset is a multimodal collection that includes radiology images and reports extracted from PMC open-access articles. This dataset features anatomical and directional concepts related to X-ray images, facilitating multilabel image classification using Unified Medical Language Systems (UMLS). The ROCO-v2 dataset is divided into three subsets: the training set, validation set, and test set. The training set contains 60,163 images, the validation set contains 9,945 images, and the test set contains 9,972 images. The intracranial hemorrhage dataset obtained from ROCO-v2 in each subset is listed in Tables **2** and **3** includes the inclusion and exclusion criteria for the images selected for the MJFSA framework-based sentiment analysis. Table **4** presents a sample of the intracranial hemorrhage dataset from ROCO-v2.

### 2.2 Brain MRI Image Enhancement through Contrast-Limited Adaptive Histogram Equalization (CLAHE)

The modified CLAHE (M-CLAHE) algorithm enhances the edges of clot regions within localized brain lobe regions, including the frontal, parietal, temporal, and occipital lobes, as well as subcortical structures, such as the cerebellum, thalamus, and basal ganglia. In the modified CLAHE algorithm, the brain MRI image is divided into tiles, and the histogram for each tile is calculated to determine the optimal clip limit based on the tile’s entropy, as shown in Fig. (**2a**). The clipped histogram is mapped into a cumulative distribution function to remap the pixel intensity and enhance the image contrast. Finally, bilinear interpolation estimates the new pixel values by calculating the average of the surrounding four pixels in a 2×2 grid in two directions, such as horizontally and vertically, and reconstructs the enhanced image, as shown in Fig. (**2b**). The procedure of brain MRI CLAHE enhancement is determined using Eq. (**1**).

**Table:** 

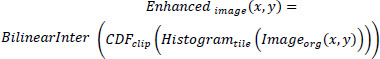	(1)

In eq. (**1**), (*x*,*y*) represents the brain CT/MRI pixel, and CDF represents the bilinear interpolation of an image. Clip limit is identified through the entropy of the tile. Image entropy is computed through Shannon entropy, as in Eq. (**2**).

**Table:** 

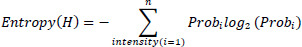	(2)

In eq. (**2**), summation indicates the sum of intensity levels in the tile.

To further enhance the visibility of anatomical structures in brain images processed by the modified CLAHE algorithm, gamma correction and image sharpening techniques are applied. In M-CLAHE, gamma correction is added to the resultant image for the enhancement of the clot regions. These enhancements are specifically targeted to improve contrast in both darker and brighter pixel regions, thereby preserving fine details and structural boundaries. The combined gamma correction and sharpening operation is defined in Eq. (**3**).

**Table:** 

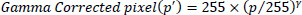	(3)

In Eq. (**3**), the gamma (γ) parameter is adaptively tuned based on the Shannon entropy of the image tile, with values ranging from 0.5 to 2.5. A γ value between 0.5 and 0.99 enhances darker regions by emphasizing lower intensity levels, thereby improving the visibility of low-signal brain areas. A γ value equal to 1 indicates no gamma correction, preserving the original image intensities. Conversely, γ values greater than 1 enhance the brightness in low-contrast regions, particularly in mid-tone gray levels. Empirical evaluation indicates that a γ value of 1.25 provides optimal enhancement for brain MRI images in this context.

Fig. (**2c**) illustrates the visualization of enhanced brain MRI images with an optimized entropy-based CLAHE model. The histogram of each tile represents the redistribution of pixel intensities after applying the entropy-based optimal clip limit. The excessive frequency of extreme intensity values in the original histogram is redistributed to preserve the local contrast of the brain image. Table **5** shows a comparison of brain MRI image enhancement using modified CLAHE, CLAHE, and Laplacian filtering methods, along with their statistical measures, including PSNR, SSIM, RMS Contrast, and Relative Contrast Change.

### 2.3 Tuned Temporal Generative Adversarial Network (Tuned T-GAN) for Generating Intracranial Hemorrhage Temporal and Spatial Images to Detect Dementia at an Earlier Stage

Tuned Temporal GAN captures changes over time during the progression of intracranial hemorrhage development [22] and is used to detect mild cognitive impairment, which leads to analysis of the progression of dementia and Alzheimer's disease in the later stages of intracranial hemorrhage [23]. The proposed Temporal GAN generates a sequence of images from a single MRI image. The Temporal GAN generates spatiotemporal images by concatenating time and spatial features from the images using 2D convolutional and ReLU layers. T-GAN generates a sequence of spatiotemporal images to enable early detection of intracranial hemorrhage and to support the prevention of dementia and Alzheimer’s disease by modeling disease progression through image generation. The T-GAN-based temporal intracranial hemorrhage model is represented in Eq. (**4**).

**Table:** 

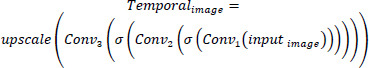	(4)

From eq. (**4**), *Conv_i_* represents the 2D convolutional layer, which takes 2-dimensional input features, such as image and time sequence information, as input. ‘*σ’* is the activation layer, and upscale is used to convert the generated image to be similar to the original input image. Fig. (**3a**) shows the architecture of T-GAN to generate the sequence of intracranial hemorrhage images to predict dementia and Alzheimer's disease. Fig. (**3b**) shows the progression of the image at different time periods.

Temporal intracranial hemorrhage images are generated using a brute-force approach and optimizing the architecture of the proposed Tuned Temporal GAN (Tuned-T-GAN). The number of convolutional layers is varied from 2 to 5, and the optimal configuration is selected based on the minimum mean squared error (MSE) between generated and original images. A five-layer configuration yields the lowest MSE, indicating high image fidelity and structural similarity. Thus, the proposed T-GAN employs five convolutional layers, as defined in Eq. (**5**).

**Table:** 

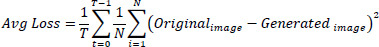	(5)

From Eq. (**5**), T indicates the time stamp, and ‘N’ indicates the total number of pixels in the image. Table **6** describes the statistical measures of the optimal number of convolutional layers and the number of filters chosen for generating temporal IH images.

Fig. (**4a** and **b**) show the statistical measure of sequential intracranial hemorrhages at different time intervals, illustrating the progression and frequency of dementia and Alzheimer’s disease over the time period. The increasing value of the mean square error indicates the progression of intracranial hemorrhages, such as the expansion of the bleeding pattern from Time 1 to Time 2. Intracranial hemorrhage is reduced due to therapeutic treatment at Time Frame 3 and Time Frame 4. Similarly, the Pearson correlation coefficient shows the linear similarity in the bleeding pattern. The increasing structural similarity from Time 0 to Time 4 indicates a reduced bleeding pattern in the intraventricular region of the brain.

### 2.4 Clinical Notes Generation for Generating Temporal Images Using Microsoft Phi-2

Microsoft Phi-2 is a small language model that generates clinical notes, such as consultation notes, progression notes, and procedure notes, from radiology text using prompts. The Microsoft Phi-2 model employs a decoder-only transformer architecture with a multi-headed self-attention mechanism to generate long-range sequential tokens of up to 2048 tokens. The model first converts the radiology text into tokens, which are then processed through self-attention layers to understand the context and relationships among the different parts of the text. Finally, the model generates concise, clinically relevant notes, approximately 700 tokens in length, including consultation notes, progression notes, and procedural notes, from the radiology text. Fig. (**5**) shows sample clinical notes generated by the small Microsoft Phi-2 language model.

### 2.5 Sentiment Classification from Clinical Narratives Using a Large Language Model

Sentiment analysis of clinical narratives involves extracting clinical judgments from various clinical notes and reports, such as consultation notes, progression notes, procedural notes, and radiology reports. This analysis aims to assess the patient's mental health state, patient experience, and support clinical decision-making [24]. Clinical narratives are evaluated using lexicon-based polarity scores, domain-specific analysis based on Unified Medical Language Systems (UMLS), and neural network-based transformer models [25]. However, conducting sentiment analysis on clinical texts is complex due to the context-dependent nature of clinical narratives and the implicit sentiments present in automated text analysis. While brain MRI images provide anatomical and pathological information, they do not convey emotional or sentimental variations. To enhance patient-informed decision support using clinical narratives, multimodal sentiment analysis is applied to both radiology reports and clinical narratives [26]. Furthermore, we have analyzed progressive and therapeutic sentiments to facilitate the early detection of intracranial hemorrhage and the prediction of dementia and Alzheimer’s disease through this multimodal sentiment analysis.

Fig. (**6**) illustrates the multimodal joint fusion sentiment analysis model (MJFSA). In MJFSA, image embeddings are extracted using the Vision Image Transformer model (ViT), while text embeddings are derived from radiology text and clinical notes using the BioBERT model. The cross-model transformer attention module receives both sets of embeddings, fusing features at an intermediate level by learning contextual information between intracranial hemorrhage MRI images and text-level features from clinical narratives. Finally, the multimodal sentiment classifier categorizes prognostic and therapeutic sentiments based on the image and clinical data. MJFSA predicts the type of intracranial hemorrhage and dementia and Alzheimer's disease, which develop over time. Fig. (**7**) shows the architecture of the ViT image embedding model.

Brain MRI images are divided into non-overlapping regions using the mathematical Eq. (**6**).

**Table:** 

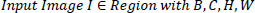	(6)

Where, B denotes the batch, C represents the number of channels, and H and W indicate the height and width of the image. Each patch is linearly projected using Eq. (**7**).

**Table:** 

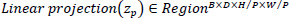	(7)

In Eq. (**7**), the patch is linearly projected across the regions with an embedding dimension (D) for each batch (B). This linear projection transforms the raw image patches into high-dimensional embeddings. Each patch embedding is normalized using Eq. (**8**).

**Table:** 

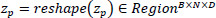	(8)

In Eq. (**8**), 'N' indicates the number of image patches in each batch, with each patch having a dimension of 'D'. Each image patch represents a semantic related to intracranial hemorrhage bleeding patterns. The positional embedding of each image is represented in Eq. (**9**).

**Table:** 

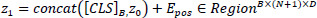	(9)

In Eq. (**9**), *z*_0_ represents the output of each embedding layer, [*CLS*]_B,_ *z*_0_ indicates the class token repeated for each image in the batch, and *E_pos_* ϵ *Region^B^*^(^*^N^*^+1) ×^*^D^* represents the learnable position embedding associated with the spatial context of the size and location of the intracranial hemorrhage for each batch. The multi-headed self-attention layer of the vision image transformer encoder model captures various types of relationships, such as bleeding patterns, the size and location of intracranial hemorrhages, and symptoms associated with dementia and Alzheimer's disease, based on the intensities of surrounding brain tissue. The multi-headed self-attention layer is represented in Eq. (**10**).

**Table:** 

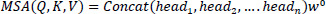	(10)

In Eq. (**10**), (Q, K, V) denotes the parameters of a learned linear projection used to obtain enhanced output. Finally, the multilayer perceptron (MLP) provides a complex structural representation of the size, shape, and texture patterns of intracranial hemorrhages. The candidate patch size is selected using the Pufferfish Optimization Algorithm (POA). Each candidate patch size is selected using Eq. (**11**).

**Table:** 

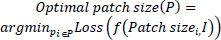	(11)

In Eq. (**11**), P represents the patch size, and I indicates the brain MRI image.

To effectively incorporate the semantic structure and anatomical location of intracranial hemorrhages within the Vision Transformer (ViT) embedding, patch sizes are dynamically optimized using the Pufferfish Optimization Algorithm (POA). The determination of the optimal patch size is formalized in Eq. (**12**).

**Table:** 

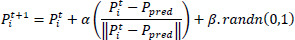	(12)

In Eq (**12**), *P^t^_i_* is the patch size of the i^th^ puffer size at ‘t’. *P_pred_* represents the worst fitness location. *α*, *β* indicates the evasion and exploration coefficient to avoid suboptimal patch size.

#### 2.5.1 Radiology Text and Clinical Notes Embedding Using the BioBERT Model

Intracranial hemorrhage and dementia-related radiology texts and clinical notes contain domain-specific terms, such as hematoma size, shape, MRI findings, and causes of cognitive impairment. To address the challenges of domain-specific sentiment analysis in radiology texts and clinical notes, the BioBERT transformer architecture model extracts complex and contextual dependencies within the text. This model predicts sentiment scores related to prognosis and therapeutic sentiments, aiding in the classification of intracranial hemorrhage subtypes and the early detection of dementia and Alzheimer's diseases. Fig. (**8**) illustrates the architecture of the BioBERT model for extracting prognosis and therapeutic sentiments to classify intracranial hemorrhage subtypes and predict dementia and Alzheimer's diseases.

In the BioBERT model, each sentence is represented as S, and the words in each sentence are tokenized using WordPiece tokenization. WordPiece tokenization converts words into subwords by finding the longest prefix of each word, recursively splitting it to effectively handle out-of-vocabulary (OOV) words. Therefore, WordPiece tokenization is represented in Eq. (**13**).

**Table:** 

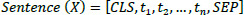	(13)

For each token, *ti* which represents the embedding, is represented in Eq. (**14**).

**Table:** 

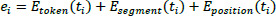	(14)

In Eq. (**14**), *E_token_* represents the token embedding, *E_segment_* represents the segment embedding and *E_position_* indicates the positional embedding.

### 2.6 Cross Attention Model (CAM)

The cross-attention score is computed through concatenating features in a shared space. The interactions between the image and text features are represented in Eq. (**15**).

**Table:** 

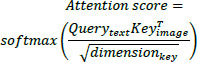	(15)

In Eq. (**15**), the keys and values depend on image patches, while the query is derived from the text. The attention mechanism computes how much each text token captures the mutual dependencies and positional embeddings from the image patches. CAM is used for multimodal classification tasks, such as prognostic and therapeutic sentiment classification, intracranial hemorrhage subtype classification, and the prediction of dementia and Alzheimer’s disease.

## 3 RESULTS AND DISCUSSION

The performance analysis of the MJFSA model is evaluated using metrics, such as precision, recall, F1-score, and accuracy. The evaluation is conducted on the ROCO-V2 dataset. Clinical emotion analysis, such as prognosis and treatment, enables physicians to evaluate disease progression, whereas sentiment analysis is based on text to understand the prognosis, treatment, and tests given by patients and physicians. Brain regions, such as the basal ganglia, lobar regions, thalamus, brainstem, and cerebellum, are linked with ICH and emotions. Indirectly, brain regions, such as the hypothalamus and anterior cingulate cortex, are affected by emotions.

The evaluation metrics are defined in Eqs. (**16**-**19**).

**Table:** 

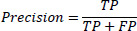	(16)

**Table:** 

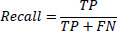	(17)

**Table:** 

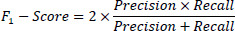	(18)

**Table:** 

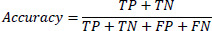	(19)

Where, TP indicates correctly predicted positive cases of intracranial hemorrhage, dementia, and Alzheimer's disease. TN represents correctly predicted negative cases of intracranial hemorrhage, dementia, and Alzheimer's disease. FP indicates incorrectly predicted positive cases of intracranial hemorrhage, dementia, and Alzheimer's disease, while FN represents incorrectly predicted negative cases of intracranial hemorrhage, dementia, and Alzheimer's disease.

Fig. (**9a** and **b**) illustrate the performance of the Joint Fusion Vision image Transformer (ViT) and BioBERT model for prognosis- and therapeutic-based sentiment analysis, respectively. Sentiment classification of intracranial hemorrhage subtypes from the ROCO-V2 dataset achieves high accuracy in identifying intraparenchymal and subdural hemorrhage using the proposed MJFSA, which leverages radiology images and contextual biomedical text.

Fig. (**10a** and **b**) illustrate the performance of the MJFSA framework in terms of training, testing, and validation accuracy curves and loss curves, respectively. It achieves smooth convergence with minimal overfitting due to the effective feature embedding of the Tuned-ViT and BioBERT models, which extract the complex long-range dependencies related to the subtype of intracranial hemorrhage, such as location, size, and shape, as well as linguistic representation through the BioBERT model in a complex neuroimaging context.

Fig. (**11a**) shows the area under the ROC curve (AUC) across all intracranial hemorrhage subtypes, demonstrating the model's ability to differentiate bleeding patterns in various regions of the brain, including the dura, arachnoid, skull, brain tissue, and ventricular system. This is achieved using a ViT-BioBERT fusion model, enhanced by domain-specific biomedical visual and textual cues.

Fig. (**11b**) presents the precision-recall curve, indicating high precision and recall for intraparenchymal and subdural hemorrhage detection. This performance is attributed to the integration of radiology images and contextual biomedical text related to brain tissue, supporting effective clinical decision-making in subtype identification and sentiment-informed treatment analysis.

Fig. (**12a** and **b**) show the comparative analysis of dementia and Alzheimer's disease prediction over time using MJFSA with the tuned ViT and BioBERT models. Intraparenchymal and subdural hemorrhages exhibit high prediction scores that gradually decrease over time, as the temporal GAN-based images simulate increasing hematoma size and texture variation compared to other intracranial hemorrhage subtypes, such as intraventricular and subarachnoid hemorrhages. This reflects outcome-based dementia prediction over time, as shown in Fig. (**12a**). Fig. (**12b**) shows that Alzheimer's disease prediction maintains a high score over time due to long-term cognitive outcomes derived from spatial imaging interpretation combined with contextual biomedical text in longitudinal neurodegenerative assessment.

Table **7** presents a summary of the performance metrics for class imbalance in IH subtypes using a temporal GAN.

To address class imbalance, synthetic spatiotemporal sequences for each IH subtype are generated using Tuned T-GAN. Fig. (**13**) shows the confusion matrix for subtype IH classification for the test set.

Prognosis and therapeutic sentiments are classified from clinical narratives and radiological texts through the early detection of intracranial hemorrhage types and the reduction of bleeding patterns and sizes at various brain locations using temporal GAN-based images. The model also predicts the progression of dementia and Alzheimer's disease. The performance analysis of early fusion, late fusion, and joint fusion strategies in MJFSA is detailed in Tables **8**-**10**, respectively. The performance evaluation of the early fusion strategy across clinical narratives and radiology reports for different IH types is presented in Table **8**. Intraparenchymal hemorrhage achieves a high prognosis sentiment classification accuracy of 86.2%, attributed to the integration of hemorrhage location with clinical narratives. Therapeutic sentiment classification yields a slightly lower accuracy of 84.7%, primarily due to treatment-related sentiment variability. This reduction is partly due to Tuned-T-GAN-generated images, which simulate bleeding patterns in brain tissues post-therapeutic intervention.

For dementia prediction, the model achieves a diagnostic accuracy of approximately 85.4% by capturing early neurodegenerative changes reflected in both textual and image textures. However, Alzheimer’s disease prediction demonstrates comparatively lower accuracy, due to interference from other overlapping cognitive disorders. The subarachnoid hemorrhage subtype shows lower performance across all classification tasks due to the diffuse nature of bleeding within the subarachnoid space. This variability complicates the identification of distinct bleeding patterns, and the early fusion strategy introduces redundant features, which reduces the model’s ability to accurately assess this subtype.

The performance evaluation of the late fusion strategy for classifying prognosis sentiment, therapeutic sentiment, and the prediction of dementia and Alzheimer’s disease over time is presented in Table **9**. Epidural intracranial hemorrhage shows a high accuracy of 82.9% for prognosis and 81.2% for therapeutic sentiment analysis, attributed to the accurate identification of the epidural bleeding pattern using combined clinical and radiological data. However, dementia and Alzheimer’s disease prediction performance is lower in cases of epidural hemorrhage compared to the subarachnoid intracranial hemorrhage subtype, due to the long-range dependencies associated with neurodegenerative changes more prevalent in subarachnoid hemorrhage cases.

Table **10** presents a performance analysis of intracranial hemorrhage subtypes, focusing on prognosis, therapeutic sentiment analysis, and the prediction of neurodegenerative disorders, such as dementia and Alzheimer's disease. This analysis employs a joint fusion strategy that integrates radiological images and text data to generate temporal T-GAN images. According to Table 10, intracranial hemorrhage subtypes achieve high precision, recall, and accuracy in clinical sentiment classification and dementia prediction over time. The joint fusion approach effectively incorporates image and text features by capturing the contextual dependencies between image patches and clinical context.

Fig. (**14**) illustrates the generated sample report, which follows the sentiment analysis of intracranial hemorrhage subtypes, as well as the detection of dementia and Alzheimer's disease over time.

Intracranial hemorrhage increases the risk of developing dementia and Alzheimer’s disease over time due to bleeding within brain tissue, which can occur in various hemorrhage types, such as intraparenchymal, subdural, subarachnoid, epidural, and intraventricular. Factors influencing dementia development in intracranial hemorrhage patients include hemorrhage size, location, expansion, and long-term cognitive impairment caused by blood clots around the hemorrhage. Therefore, the prediction of dementia and Alzheimer’s disease in intracranial hemorrhage patients is analyzed using brain MRI scans. Radiologists analyze these scans and provide reports detailing the findings, impressions, hematoma size, and locations of bleeding patterns. Table **11** summarizes state-of-the-art techniques for the early detection of dementia and Alzheimer’s disease, along with their disadvantages.

Hence, traditional techniques provide less sensitivity in the identification of early dementia in specific regions. Visual rating scales vary and are subject to inter-rater variability due to the complex bleeding patterns of intracranial hemorrhage subtypes. These methods are computationally expensive and require expert interpretation. Therefore, we applied prognosis and therapeutic sentiment analysis using deep learning and large language models for intracranial hemorrhage–based dementia and Alzheimer’s disease detection.

The proposed MJFSA model analyzes the gap between radiological observations and cognitive outcomes to evaluate prognosis and therapeutic sentiments associated with intracranial hemorrhage and dementia related to Alzheimer’s disease. The model identifies positive prognosis and therapeutic sentiments based on lesion progression, therapy response, and clinical impressions. Conversely, negative sentiment polarity is detected through worsening microbleeds and ventricular enlargement linked to neurodegenerative impairments.

Lesion progression, microbleed patterns, and ventricular enlargement are analyzed using the deep learning model. Thus, integrating the Multimodal Joint Fusion Sentiment Analysis (MJFSA) model improves the detection accuracy for intracranial hemorrhage patients with dementia and Alzheimer’s disease.

Table **12** presents state-of-the-art techniques for the early detection of intracranial hemorrhage subtypes and the prevention of dementia and Alzheimer’s disease. Table **13** shows the ablation analysis of the proposed MJFSA model, while sample prognosis sentiment analysis and therapeutic sentiment analysis reports generated using the MJFSA framework are presented in Table **14**.

## 4 LIMITATIONS

The proposed multimodal architecture achieves high accuracy in the early detection of intracranial hemorrhages and the prediction of dementia and Alzheimer's disease over time through prognosis and therapeutic sentiment analysis reports. However, the synthetic data generation using Tuned-Temporal GAN and Microsoft-Phi-2 introduces artifacts in clinical environments, affecting the generalizability of pathological findings. The generation of reports results in a lack of contextual depth in traditional records, creating a risk of overfitting in various medical domains. The integration of Tuned ViT, clinical BioBERT, and the GAN model increases computational complexity, making the deployment of the proposed MJFSA model challenging in resource-constrained environments. Table **15** illustrates the adaptation of the MJFSA framework to a diverse population with different imaging modalities, including CT and PET.

A summary of the real-time implementation of the MJFSA framework, based on the effects of radiologist workload and patient outcomes, is presented in Table **16**.

To address computational complexity, temporal images are preprocessed and compressed using pruned and quantized CNN architectures. The TinyBERT model is used for radiological clinical narrative reports. Prognosis and therapeutic sentiment classifiers are implemented on Raspberry Pi hardware units, effectively reducing overall computational demands. Fig. (**15**) illustrates the flowchart for reducing computational complexity using a Raspberry Pi hardware unit.

An increasing number of false positives and false negatives leads to unnecessary diagnostic procedures, patient anxiety, and an increased clinical workload for physicians and radiologists in the hospital. To solve the above limitations, by integrating historical radiology reports with newly acquired samples, the MJFSA framework generates temporal imaging sequences accompanied by structured clinical documentation, including clinical notes, procedural reports, and progressive notes related to intracranial hemorrhages and their classifications. Through the application of sentiment analysis to these narratives, the system enhances its ability to detect dementia and Alzheimer’s disease early. This integrative approach not only enhances diagnostic accuracy but also contributes to mitigating neurocognitive decline through timely intervention. Moreover, the incorporation of real-world pilot implementations within clinical environments has demonstrated promising potential for increasing recovery rates and reducing associated mortality, thereby reinforcing the framework’s practical utility in early-stage neurological assessments.

## CONCLUSION

The proposed MJFSA framework analyzes prognosis and therapeutic sentiment analysis based on radiological images and clinical narratives, including radiology texts, progression notes, consultation notes, and clinical narratives of patients with intracranial hemorrhage. The proposed model considers the effects of dementia and Alzheimer's disease related to the bleeding patterns in brain tissue. The proposed MJFSA model combines Tuned ViT and BioBERT to generate temporal images and narrative reports, enhancing image diagnostics through early detection of cognitive decline. The model demonstrates high accuracy in identifying different subtypes of intracranial hemorrhage, such as intraparenchymal, subdural, and epidural, while facilitating early detection of dementia and Alzheimer's disease through multimodal and joint feature fusion, and achieves cognitive outcomes in clinical decision support systems by integrating visual and textual data. However, the proposed model has limitations, including the introduction of image and data artifacts, as well as high computational requirements. In the future, the model is intended to support long-term monitoring of intracranial hemorrhage patients to aid in the prevention of dementia and Alzheimer’s disease as part of routine clinical diagnostics.

## AUTHORS' CONTRIBUTIONS

The authors confirm their contribution to the paper as follows: study conception and design: K.Balasaranya and P.Ezhumalai; data collection, analysis, and interpretation of results: P.Ezhumalai; draft manuscript: K.Balasaranya, P.Ezhumalai, and N.R.Shanker. All authors reviewed the results and approved the final version of the manuscript.

## Figures and Tables

**Fig. (1a):**
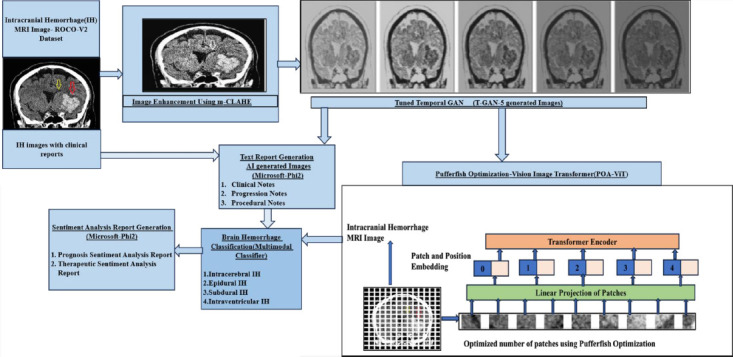
Architecture of multimodal sentiment analysis using multi-joint fusion sentiment analysis model (MJFSA).

**Fig. (1b):**
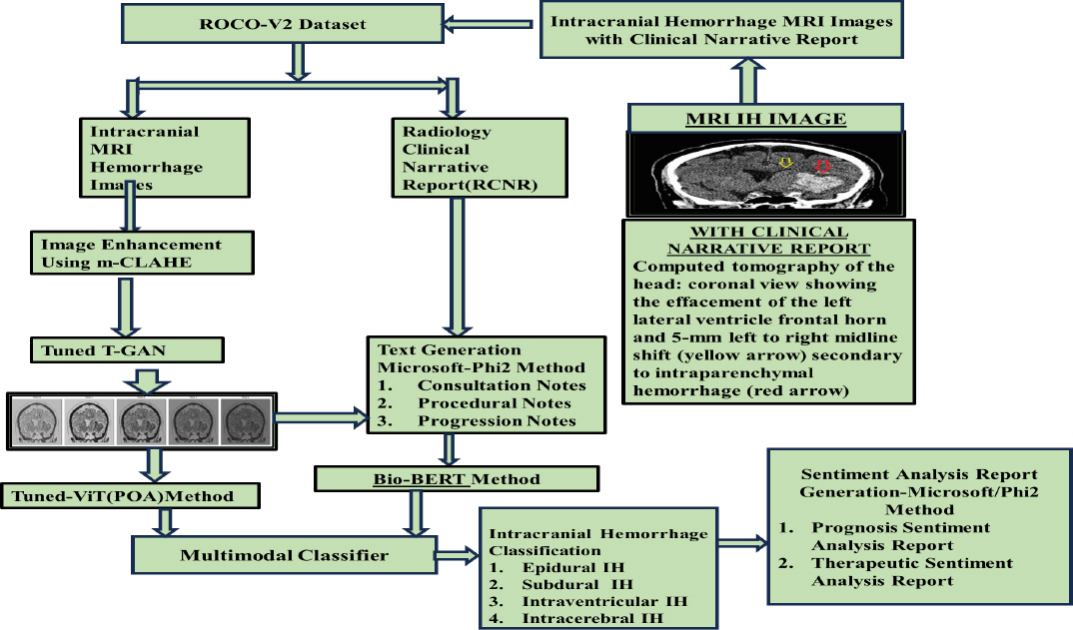
Flow diagram of prognosis and therapeutic-based sentiment analysis from clinical narrative reports of ih subtype classification and prediction of dementia and Alzheimer's Disease.

**Fig. (2a):**
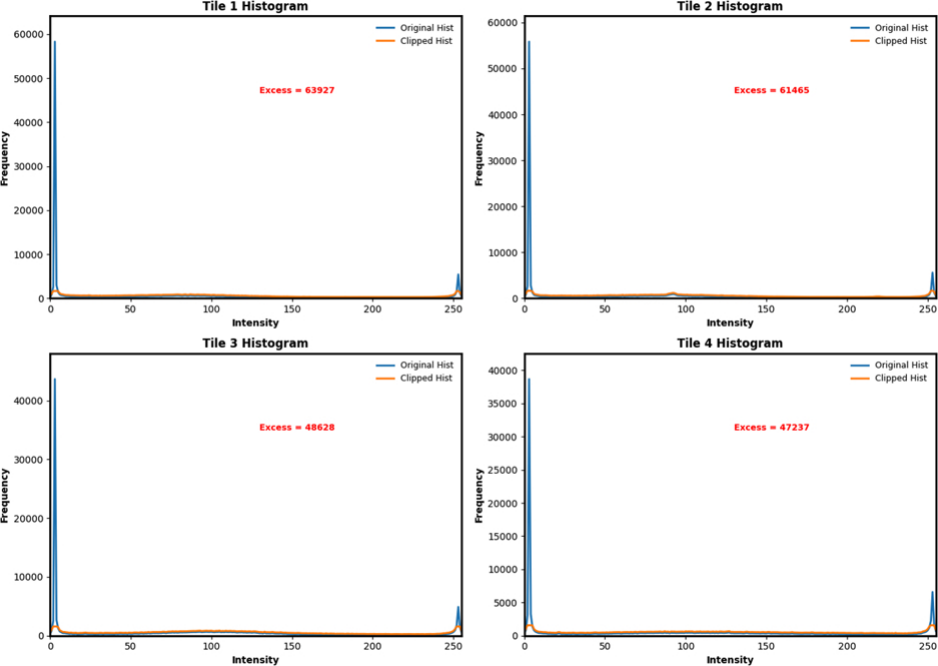
Histogram of tiles.

**Fig. (2b):**
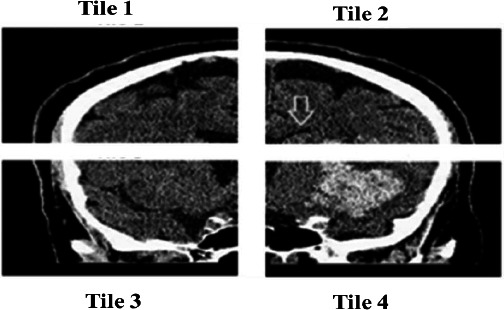
Enhance image quality by dividing into 4 equal tiles.

**Fig. (2c):**
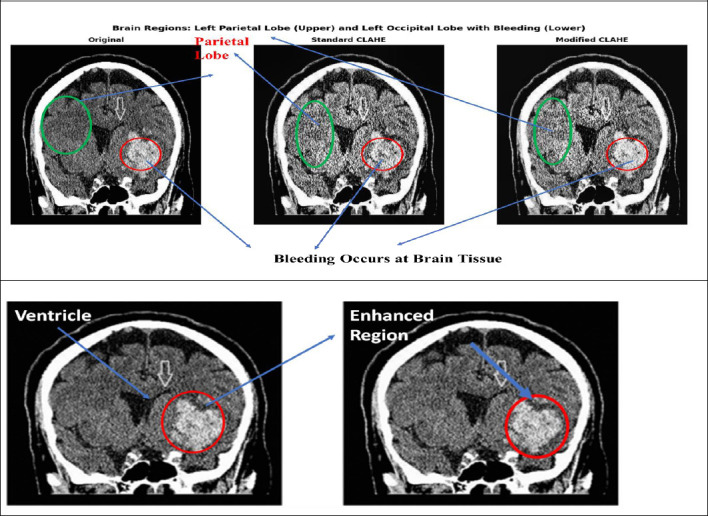
Brain Image Enhancement Using CLAHE.

**Fig. (3a):**
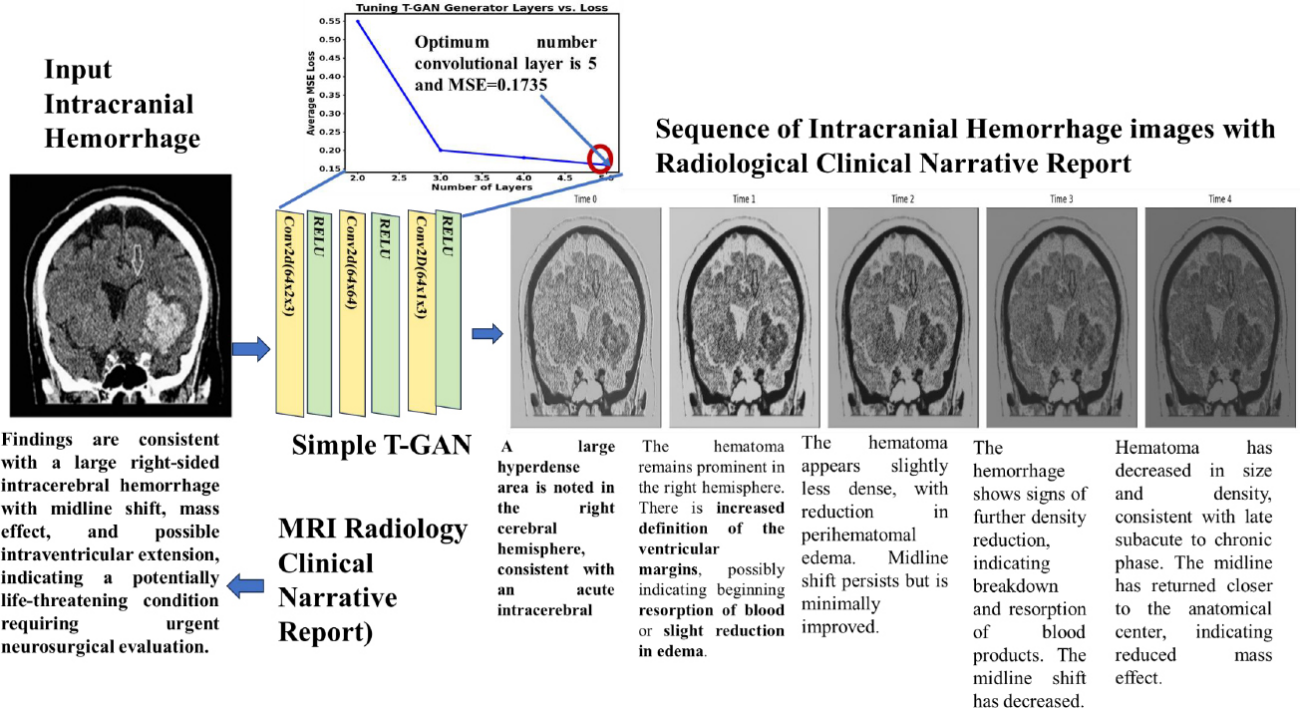
Architecture of Tuned T-GAN.

**Fig. (3b):**
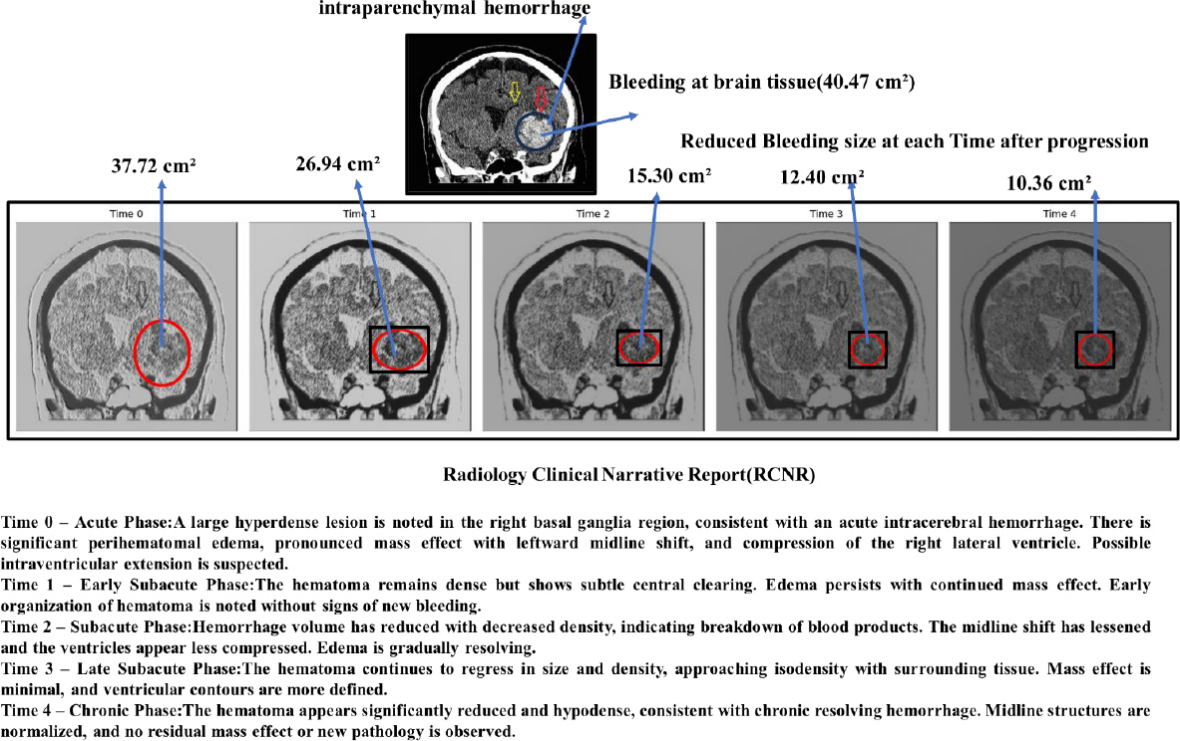
Progression of image at different time periods.

**Fig. (4a,b):**
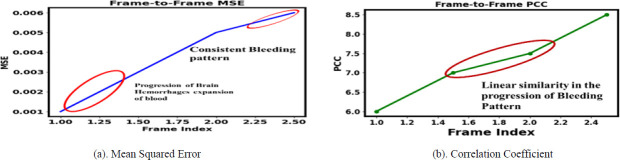
Statistical measure of sequential intracranial hemorrhages using T-GAN at different time periods of progression and development of dementia and alzheimer's diseases.

**Fig. (5):**
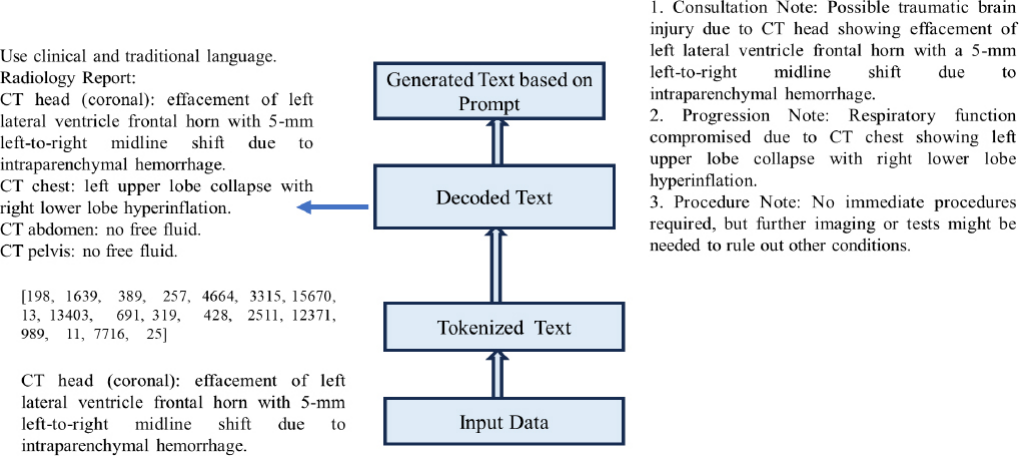
Clinical notes generated using microsoft Phi-2 for generated temporal images.

**Fig. (6):**
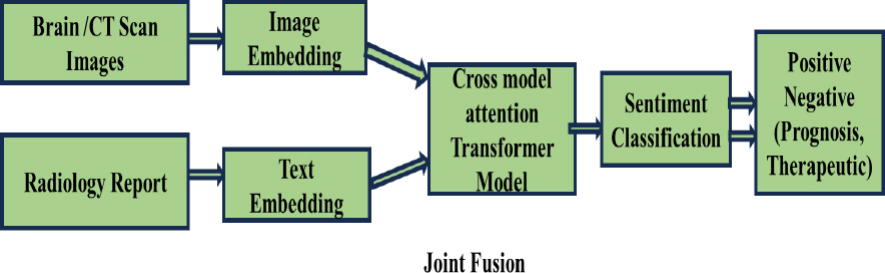
Multimodal joint fusion sentiment analysis model for intracranial hemorrhage detection and prediction of dementia and alzheimer's diseases (MJFSA).

**Fig. (7):**
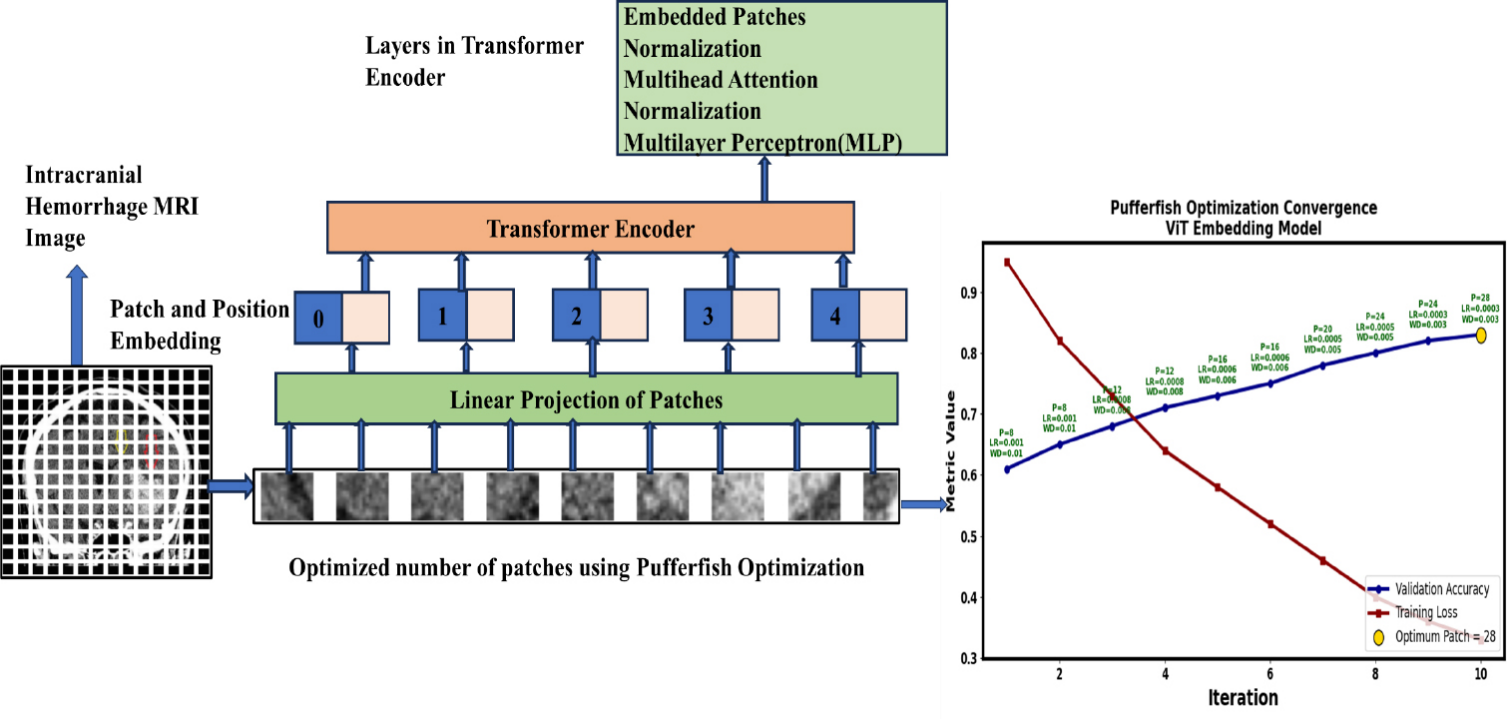
Architecture of Tuned ViT model for image patch embedding.

**Fig. (8):**
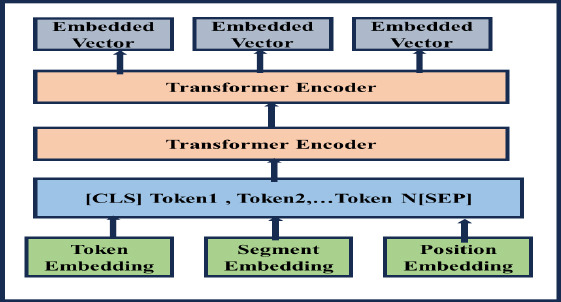
Architecture of the BioBERT model for generating text embedding for prognosis- and therapeutic-based sentiment analysis.

**Fig. (9):**
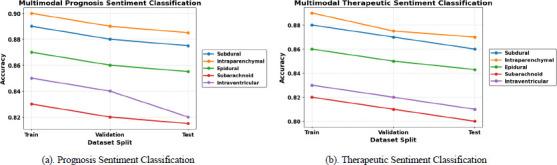
(**a**) Prognosis and (**b**) Therapeutic Sentiment Classification for Intracranial Hemorrhage Subtype Using DL and LLM.

**Fig. (10):**
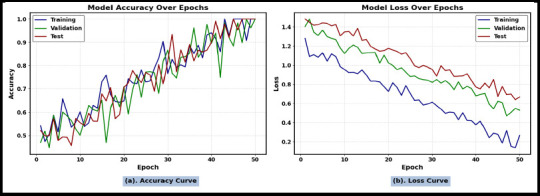
(**a**) Accuracy Curve Showing the Performance of the Proposed MJFSA Model in Classifying Intracranial Hemorrhage Subtypes Using the ROCO-V2 Dataset. (**b**). Loss Curve Using DL and LLM Models.

**Fig. (11):**
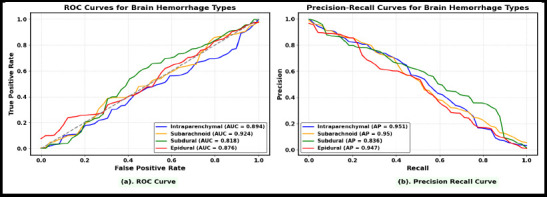
(**a**). ROC Curve for Intracranial Hemorrhage Subtype Classification Using the ROCO-V2 Dataset. 11 (**b**) Precision–Recall Curve Demonstrating the Performance of DL and LLM Models.

**Fig. (12):**
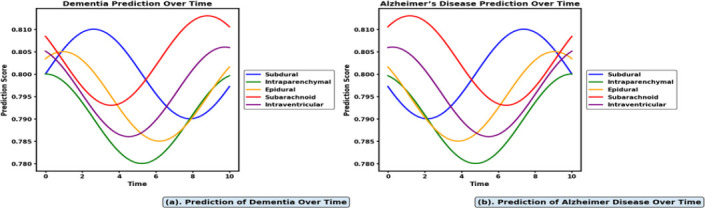
(a) Dementia prediction over time due to IH. (b). Alzheimer's Prediction over time due to IH.

**Fig. (13):**
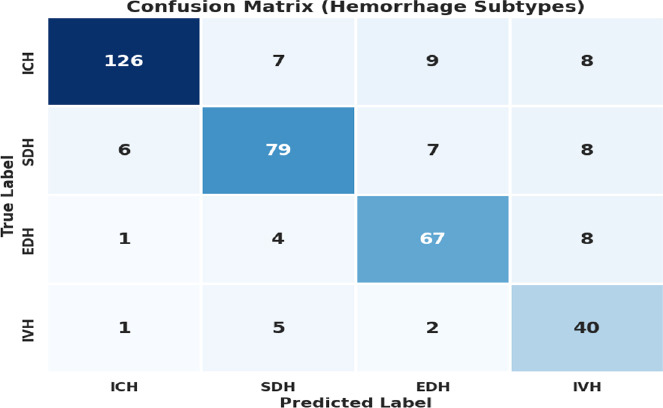
Confusion matrix for IH subtype classification on the test set.

**Fig. (14):**
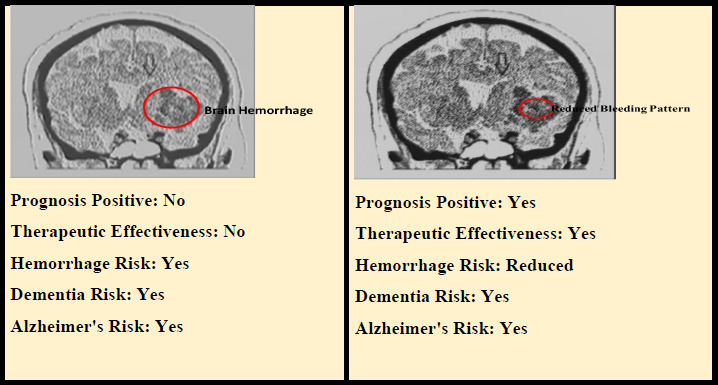
Sample report generated from brain MRI and RCNR using MJFSA.

**Fig. (15):**
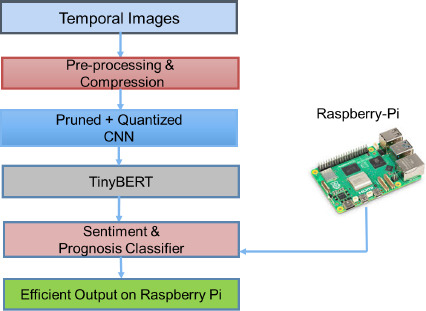
Flow chart to reduce computational complexity through Raspberry Pi.

**Table 1:** Hemorrhage types and risk of dementia or Alzheimer’s Disease.

**Hemorrhage Type**	**Risk of Dementia / Alzheimer's**	**Relationship between IH/Dementia and Alzheimer’s**
**Intracerebral Hemorrhage (ICH)**	**High**	Strongly associated with vascular dementia due to direct brain tissue damage.
**Subdural Hemorrhage (SDH)**	**Moderate**	Chronic SDH, especially in the elderly, can cause prolonged pressure and cognitive decline.
**Epidural Hemorrhage (EDH)**	**Low**	Typically occurs in younger individuals with trauma; it has less long-term cognitive impact if treated promptly.
**Intraventricular Hemorrhage (IVH)**	**Moderate to High**	Associated with cognitive impairment, especially in premature infants or when secondary to ICH.

**Table 2:** Intracranial hemorrhage dataset obtained from the ROCO-V2.

**Dataset Split**	**Hemorrhage Subtype**	**Number of Cases**	**Images Generated (10 per case)**
**Train**	Intracerebral Hemorrhage (ICH)	142	1,420
	Subdural Hemorrhage (SDH)	91	910
	Extradural Hemorrhage (EDH)	58	580
	Intraventricular Hemorrhage (IVH)	49	490
	Mixed or Unspecified Types	38	380
	**Total (Train)**	**378**	**3,780**
**Validation**	Intracerebral Hemorrhage (ICH)	26	260
	Subdural Hemorrhage (SDH)	17	170
	Extradural Hemorrhage (EDH)	11	110
	Intraventricular Hemorrhage (IVH)	9	90
	Mixed or Unspecified Types	10	100
	**Total (Validation)**	**73**	**730**
**Test**	Intracerebral Hemorrhage (ICH)	28	280
	Subdural Hemorrhage (SDH)	18	180
	Extradural Hemorrhage (EDH)	12	120
	Intraventricular Hemorrhage (IVH)	10	100
	Mixed or Unspecified Types	7	70
	**Total (Test)**	**75**	**750**
	**Grand Total**	**526**	**5,260**

**Table 3:** Inclusion and exclusion criteria for the dataset based on physician opinion.

**Criteria Type**	**Criteria**	**Remarks**	**Physician Opinion**
Inclusion	Availability of Clinical Narrative Report (RCNR)	Enables joint image-text fusion using the MJFSA framework.	Required for context-aware diagnostic interpretation.
	Complete and Readable DICOM MRI Data	Ensures valid image sequences and metadata for processing.	Essential for reliable clinical evaluation and structured review.
	MRI Resolution ≥ 128×128 pixels	Provides adequate detail for enhancement, segmentation, and feature extraction.	Minimum resolution threshold to ensure anatomical fidelity.
	Axial Plane MRI Slices	Consistent with the model’s training dataset and orientation requirements.	Axial slices provide a standardized anatomical comparison.
	Digitally Typed and Structured RCNR Text	Ensures compatibility with NLP processing tools like Bio-BERT.	Structured narratives reflect clinical workflow and ensure interpretability.
Exclusion	Missing Clinical Narrative Report (RCNR)	The fusion pipeline requires both image and text for joint multimodal analysis.	Lack of text limits contextual diagnosis and undermines fusion integrity.
	Corrupted or Incomplete MRI Data	DICOM headers missing or sequences unreadable; not suitable for processing.	Non-diagnostic quality; clinical review not possible.
	Low-Resolution MRI (<128×128 pixels)	Image resolution is insufficient for the enhancement and feature extraction stages.	The image lacks sufficient anatomical clarity for accurate diagnosis.
	Non-Axial Imaging Plane Used	The model is trained exclusively on axial MRI slices.	Inconsistency may introduce diagnostic variation; re-acquisition is advised.
	Handwritten or Unstructured RCNR (OCR Failure)	OCR failed; Bio-BERT text processing cannot proceed.	Non-standard documentation; not suitable for automated NLP analysis.

**Table 4:** Sample intracranial hemorrhage images from the ROCO-V2 Dataset.

Images/Radiology Report (Clinical Narrative)	Temporal Images-Generative Adversarial Network (T-GAN)
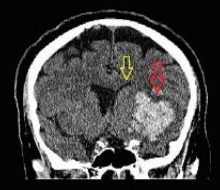 Computed tomography of the head: coronal view showing the effacement of the left lateral ventricle, frontal horn, and 5-mm left to right midline shift (yellow arrow) secondary to intraparenchymal hemorrhage (red arrow)	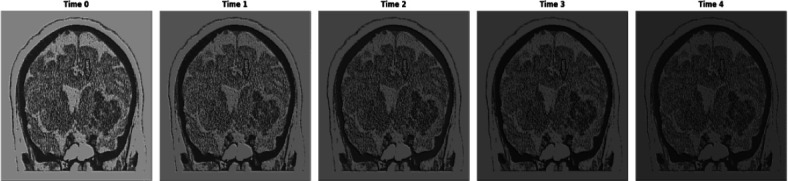 Findings: Serial coronal CT images demonstrate a progressive resolution of a previously identified right basal ganglia hemorrhage. At Time 0, a well-circumscribed hyperdense lesion with surrounding edema and mild mass effect is visualized. As the timeline progresses from Time 1 to Time 4, there is a steady decrease in hematoma density and volume, with significant resolution of mass effect and peri hematom al edema. The ventricular anatomy becomes more defined, and midline structures return to near-normal alignment. No new hemorrhagic events, hydrocephalus, or ischemic changes are observed. The imaging appearance at Time 4 suggests the hematoma has entered a chronic phase, characterized by hypodensity and near-complete resorption. Impression: Progressive resolution of right basal ganglia intracerebral hemorrhage over the observed interval. No evidence of rebleeding or secondary complications. Current findings are consistent with late subacute to chronic hemorrhage stage. Recommendation: Continue clinical monitoring. Optional MRI may be considered to assess gliosis or underlying structural changes, if clinically warranted.
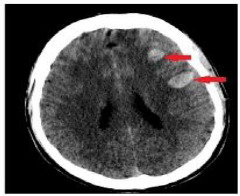 Axial CT scan without contrast enhancement. Red arrows show two spots of unilateral typical deep cortical venous hemorrhages with minor surrounding oedema in the left frontal lobe of the brain. The frontal hemorrhage spot crosses the borders of the arterial brain vascular territories.	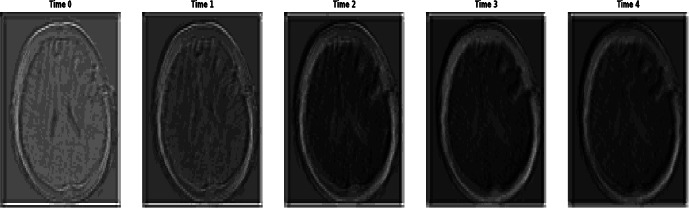 Serial axial CT images show a hyperdense area within the right frontoparietal region, consistent with acute intracerebral hemorrhage or hemorrhagic transformation of infarct at Time 0. The lesion demonstrates well- defined margins with associated surrounding hypo attenuation, indicative of vasogenic edema. Over successive time points (Time 1 to Time 4), there is a gradual reduction in the hyper density, signifying hematoma resorption. The surrounding edema also reduces, and there is no progression of mass effect or ventricular compression. The sukal spaces and adjacent cortical structures appear to retum toward normal. By Time 4, the hematoma appears hypodense, consistent with the chronic phase of hemorrhage resolution. No new lesions, midline shift, or hydrocephalus are seen. Impression: Evolution of a right frontoparietal intracerebral hemorrhage from acute to chronic phase with marked resolution. No imaging evidence of rebleeding, infarct progression, or hydrocephalus. Stable follow-up without new conceming features. Recommendation: Continue clinical monitoring. MRI with GRE/SWI sequences may be beneficial if further evaluation of hemosiderin deposition or residual gliosis is needed.

**Table 5:** Statistical performance analysis of brain MRI image enhancement.

**Image ID**	**mCLAHE**	**CLAHE**	**Laplacian**
**ROCOv2_2023_test_000020** 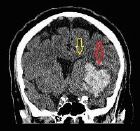	PSNR: 35.35 dB Entropy: 5.7457 → 5.7623 SSIM: 0.9975 RMS Contrast: 1.1426 → 1.1204 Relative Contrast Change: -0.0194 **Interpretation**: mCLAHE gave superior results due to entropy-based tile and gamma correction. Structural detail and similarity were well-preserved.	PSNR: 33.12 dB Entropy: 5.7457 → 5.7510 SSIM: 0.9851 RMS Contrast: 1.1426 → 1.1102 Relative Contrast Change: -0.0283 **Interpretation**: Moderate enhancement, with slightly reduced structural preservation.	PSNR: 30.87 dB Entropy: 5.7457 → 5.7492 SSIM: 0.9724 RMS Contrast: 1.1426 → 1.1078 Relative Contrast Change: -0.0305 **Interpretation**: Least effective; lower quality and contrast retention compared to mCLAHE.
**ROCOv2_2023_test_000100** 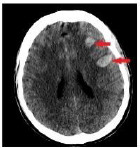	PSNR: 36.10 dB Entropy: 5.6834 → 5.7018 SSIM: 0.9980 RMS Contrast: 1.1985 → 1.1802 Relative Contrast Change: -0.0153 **Interpretation**: Excellent clarity and detail retention with minimal distortion. mCLAHE again outperformed others.	PSNR: 33.89 dB Entropy: 5.6834 → 5.6885 SSIM: 0.9860 RMS Contrast: 1.1985 → 1.1604 Relative Contrast Change: -0.0318 **Interpretation**: Moderate image improvement, but slightly less sharp than mCLAHE.	PSNR: 31.56 dB Entropy: 5.6834 → 5.6850 SSIM: 0.9741 RMS Contrast: 1.1985 → 1.1563 Relative Contrast Change: -0.0352 **Interpretation**: Underperformed; lower PSNR and SSIM imply detail loss and weaker enhancement.
**ROCOv2_2023_test_000577** 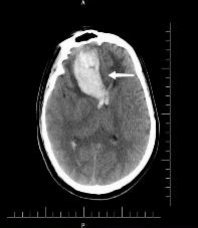	PSNR: 35.90 dB Entropy: 5.8022 → 5.8286 SSIM: 0.9972 RMS Contrast: 1.1267 → 1.1055 Relative Contrast Change: -0.0188 **Interpretation**: mCLAHE produced high-fidelity enhancement with clear structural features retained.	PSNR: 33.15 dB Entropy: 5.8022 → 5.8084 SSIM: 0.9842 RMS Contrast: 1.1267 → 1.0928 Relative Contrast Change: -0.0301 **Interpretation**: Acceptable clarity, but lower detail retention than mCLAHE.	PSNR: 30.43 dB Entropy: 5.8022 → 5.8049 SSIM: 0.9710 RMS Contrast: 1.1267 → 1.0901 Relative Contrast Change: -0.0325 **Interpretation**: Performance was poorest; loss of contrast and finer image details observed.

**Table 6:** Statistical measure for choosing 5 convolutional layers and the number of filters.

**Model**	**# Conv Layers**	**MSE ↓**	**SSIM ↑**	**PSNR (dB) ↑**	**Dice ↑**	**Radiologist Score (1–5) ↑**	**Interpretation**
Shallow GAN	2,32	0.0215	0.78	23.9	0.68	2.6	Loss of anatomical detail
Intermediate GAN	3,64	0.0173	0.82	25.6	0.72	3.2	Improved texture, but missing subtle changes
Deep GAN	4,128	0.0141	0.86	27.1	0.77	3.9	Good spatial definition
**Tuned-T-GAN (Ours)**	**5,128**	**0.0116**	**0.89**	**28.4**	**0.82**	**4.6**	Optimal fidelity, structure, and realism
U-Net + RNN	N/A	0.0152	0.84	26.2	0.75	3.7	Slight temporal flicker
3D GAN	N/A	0.0128	0.87	27.5	0.79	4.2	High cost, marginal gain

**Table 7:** Classification results for IH subtypes after temporal GAN augmentation.

**Class**	**Precision**	**Recall**	**F1-Score**
ICH	0.92	0.91	0.91
SDH	0.88	0.90	0.89
EDH	0.86	0.88	0.87
IVH	0.89	0.86	0.87

**Table 8:** Performance analysis of early fusion in MJFSA.

**Intracranial hemorrhage Subtype**	**Sentiment Classification/Dementia Prediction**	**Accuracy (%)**	**Precision (%)**	**Recall (%)**	**F1-Score (%)**	**AUC (%)**
Intraparenchymal	Prognosis Sentiment Classification	86.2	84.1	85.0	84.5	88.7
	Therapeutic Sentiment Classification	84.7	82.5	83.9	83.2	87.2
	Dementia Prediction	85.4	83.8	84.3	84.0	88.0
	Alzheimer’s Disease Prediction	84.2	82.1	83.0	82.5	86.3
Subarachnoid	Prognosis Sentiment Classification	83.6	81.5	82.0	81.7	86.4
	Therapeutic Sentiment Classification	82.1	80.3	80.9	80.6	85.1
	Dementia Prediction	83.3	81.9	82.4	82.1	86.5
	Alzheimer’s Disease Prediction	81.5	79.6	80.5	80.0	84.7

**Table 9:** Performance analysis of late fusion in MJFSA.

**Intracranial hemorrhage Subtype**	**Sentiment Classification/Dementia Prediction**	**Accuracy (%)**	**Precision (%)**	**Recall (%)**	**F1-Score (%)**	**AUC (%)**
Epidural	Prognosis Sentiment Classification	82.9	81.0	81.7	81.3	85.4
	Therapeutic Sentiment Classification	81.2	79.1	80.0	79.5	83.9
	Dementia Prediction	80.5	78.7	79.6	79.1	84.2
	Alzheimer’s Disease Prediction	79.4	77.5	78.3	77.9	82.6
Subarachnoid	Prognosis Sentiment Classification	81.6	79.8	80.4	80.1	83.7
	Therapeutic Sentiment Classification	80.3	78.4	79.0	78.7	82.9
	Dementia Prediction	81.1	79.3	80.0	79.6	84.0
	Alzheimer’s Disease Prediction	78.9	77.0	77.7	77.3	82.1

**Table 10:** Performance analysis of joint fusion in MJFSA.

**Intracranial hemorrhage Subtype**	**Sentiment Classification/Dementia Prediction**	**Accuracy (%)**	**Precision (%)**	**Recall (%)**	**F1-Score (%)**	**AUC (%)**
Subdural	Prognosis Sentiment Classification	94.4	88.0	88.6	88.3	91.2
	Therapeutic Sentiment Classification	96.1	86.7	87.2	86.9	90.1
	Dementia Prediction	95.0	87.8	88.4	88.1	91.0
	Alzheimer’s Disease Prediction	92.6	86.2	86.8	86.5	89.7
Intraparenchymal	Prognosis Sentiment Classification	95.2	89.0	89.7	89.3	92.3
	Therapeutic Sentiment Classification	96.9	87.3	88.0	87.6	90.9
	Dementia Prediction	94.8	88.5	89.1	88.8	91.6
	Alzheimer’s Disease Prediction	95.3	86.9	87.6	87.2	90.4

**Table 11:** State-of-the-art techniques for the early detection of dementia and Alzheimer's Disease.

**Reference**	**Type Dementia**	**Imaging**	**Technique Used**	**Dementia Location**	**Disadvantage**
[27]	Vascular dementia	Ultrasound	Cerebral blood flow	Subcortical white matter, basal ganglia, thalamus	Sensitive to small vessel disease
[28]	Behavioral and semantic variants of frontotemporal dementia	MRI	Visual rating scales	Frontal lobes (behavioral variant), anterior temporal lobes (semantic variant)	Subjective interpretation
[29]	Alzheimer’s disease	MRI	Transfer learning with multiclass MRI scans	Medial temporal lobes (hippocampus, entorhinal cortex, parahippocampal gyrus)	Computational cost is high
[30]	Various dementias (focus on blood-brain barrier pathology)	Advanced imaging (*e.g*., DCE-MRI, PET)	Blood-brain	Include hippocampus, cortex	High cost

**Table 12:** State-of-the-art techniques for the early detection of intracranial hemorrhage subtypes and the prevention of dementia and Alzheimer’s disease.

**Reference**	**Techniques Used**	**Accuracy / Precision / Recall (Intracranial Hemorrhage and Dementia)**	**Types of Intracranial Hemorrhage**	**Disadvantage**
[31]	U-Net	Accuracy: 91.8% Precision: 92.5% Recall: 92%	Epidural, subdural, subarachnoid, intraparenchymal, intraventricular	Leads to overfitting
[32]	Deep learning framework (CNN) for CT brain images	Accuracy: 94% Precision: 93% Recall: 92%	Intracranial hemorrhage	Requires a large dataset
[33]	CNN architecture for MR image analysis	Accuracy: 93% Precision: 92% Recall: 91%	Ischemic stroke, hemorrhagic stroke	Difficult to interpret small lesions
[34, 35]	CT and MRI structural analysis	Accuracy:89% Precision:88% Recall:88.4%	Intracranial malformations, hemorrhagic lesions in children	Depends on expert interpretation
**Proposed**	**Radiology Text, Clinical Notes, and Brain MRI Images(MJFSA)**	**Accuracy:96.5%** **Precision:94.5%** **Recall: 95.6%**	**Epidural, subdural, subarachnoid, intraparenchymal, intraventricular**	**The proposed framework extracts the contextual and longitudinal dependencies between the image and text.**

**Table 13:** Ablation analysis of proposed mjfsa model for sentiment-based intracranial hemorrhage detection and risk prediction of dementia/Alzheimer's disease.

**Model**	**Prognosis Accuracy (%)**	**Therapeutic Sentiment Accuracy (%)**	**Dementia/Alzheimer Risk Prediction Accuracy (%)**	**Remarks**
Only Clinical BioBERT (Text Encoder)	78.3	76.5	72.1	Text-only clinical BERT model.
+ CLAHE Image Enhancement	80.1	78.2	74.8	Contrast enhancement improves the performance of multimodal analysis.
+ T-GAN Generated Images	82.6	79.3	76.9	Synthetic temporal features added for early-stage hemorrhage detection.
+ ViT Image Encoder	85.4	81.7	79.2	Deep visual context modeling increases the performance.
+ Image & Text Alignment	88.0	83.9	82.6	Alignment of modalities enables better context understanding.
+ Feature Fusion Module	90.2	86.1	85.7	Fused features provide stronger shared representations across modalities.
+ GAN (Phi2) Generated Clinical Notes	91.6	88.0	87.3	Augmented clinical narratives enrich text diversity and context.
**Full Model**	**96.5**	**94.5**	**95.6**	**Best performance achieved with the full proposed architecture and multimodal input.**

**Table 14:** Sample prognosis sentiment analysis and therapeutic sentiment analysis reports generated using the MJFSA framework for intracranial hemorrhage types and risk of dementia or Alzheimer’s Disease.

**Hemorrhage Type/** **Patient ID**	**Risk of Dementia / Alzheimer’s** **(Image analysis)**	**Estimated Association –(EA) (%)** **(Clinical native report text analysis)** EA= (Number of Complex Words) / (Total Number of Words) * 100	**Prognosis Sentiment Analysis Report** **(proposed MJFSA)**	**Therapeutic Sentiment Analysis Report** **(proposed MJFSA)**
**Intracerebral Hemorrhage (ICH)** **Patient ID-25**	**High**	21%	**Guarded to poor**, especially if large or deep (*e.g*., basal ganglia, thalamus). High risk of long-term disability and recurrent strokes.	**Negative-Biased**: Limited treatments for reversing damage; focus is on blood pressure control, rehab, and stroke prevention. High neurological burden.
**Subdural Hemorrhage (SDH)** **Patient ID-37**	**Moderate**	12%	**Variable**: Chronic SDH in the elderly has better outcomes if treated early (via burr hole). Acute SDH can be life-threatening.	**Neutral to Positive**: Surgical drainage is effective in chronic cases. Risk of recurrence, but cognitive recovery is possible. Requires careful follow-up.
**Extradural Hemorrhage (EDH)** **Patient ID-42**	**Low**	<2%	**Good if treated early**. Lucid interval helps in early diagnosis. Often, there is a full recovery in younger patients.	**Positive**: Surgical evacuation (craniotomy) is typically curative. Favorable prognosis with timely intervention. Low association with chronic cognitive decline.
**Intraventricular Hemorrhage (IVH)** **Patient ID-59**	**Moderate to High**	18%	**Poor to moderate,** depending on cause and extent. It can lead to hydrocephalus, increased ICP, or neurodevelopmental delay in infants.	**Cautiously Negative**: Limited therapeutic options beyond supportive care, CSF diversion (*e.g*., ventriculostomy), and neuroprotection. Cognitive risks are high in neonates.

**Table 15:** MJFSA framework adaptation on diverse population and imaging modality.

**Adaptation**	**Current MRI-Based MJFSA**	**CT Modality (Adaptation Required)**	**PET Modality (Adaptation Required)**	**Pediatric/Geriatric Population (Adaptation Required)**
**Image Input Format**	High-resolution axial MRI (ROCO-V2)	CT slices (often lower soft-tissue contrast)	Functional metabolic images, lower spatial detail	Varying anatomy & resolution needs
**Image Enhancement**	m-CLAHE tailored for MRI contrast	CT requires different contrast-specific preprocessing	Noise suppression and normalization methods are required	Adaptive enhancement for small-sized brains in pediatrics
**Temporal Image Generation**	Tuned T-GAN trained on MRI data	Retraining needed on the CT sequence data	Not directly applicable; alternate models like sequence GANs needed	Must reflect age-dependent brain structure variation
**Text Generation (Phi2)**	MRI-based RCNR generation using common terms	CT terms differ; the model must be fine-tuned for the radiologist's CT language	PET reports emphasize uptake and metabolic rates	Pediatric reports use developmental context; geriatrics cite comorbidity
**ViT Model**	Tuned-ViT trained on structural features of MRI	Retraining required for intensity pattern differences in CT	Requires feature extraction methods compatible with PET resolution	ViT must learn age-specific morphology
**Text Encoder (Bio-BERT)**	Trained on standard adult radiological language	Should be re-trained with the CT-based RCNR corpus	Needs training with PET-specific vocabulary	Add training corpora for pediatric and geriatric report structures
**Classification Output**	ICH, EDH, SDH, IVH, based on MRI features	CT visible hemorrhages might differ in appearance and detection timing	PET unlikely to directly classify hemorrhage; may aid in prognosis	Needs adjustment for pediatric-specific hemorrhage (*e.g*., birth trauma)
**Sentiment Analysis Utility**	Prognosis and therapeutic sentiment tailored to the MRI narrative	CT-based prognosis differs in acute care settings	PET prognosis focuses on the metabolic response to injury	Consider age-adjusted therapy language and comorbidities

**Table 16:** Real-time diagnostic effects on radiologist workload and patient outcomes.

**Workflow Stage**	**Traditional Radiologist Role (Manual)**	**Proposed Framework (Automated/Assisted)**	**Impact on Radiologist Workload**	**Impact on Patient Outcome**
Initial Image Quality Check	Radiologist inspects image sequences for clarity, completeness, and plane orientation.	Image enhancement using m-CLAHE ensures consistent input quality.	Reduced need for quality rechecks.	Fewer scan repetitions, faster progression to diagnosis.
Image Review and Lesion Identification	Manually detects and annotates hemorrhagic regions across slices.	Tuned T-GAN generates synthetic temporal sequences, enhancing visibility and coverage.	Decreases effort in lesion tracking.	Improved lesion visibility across time enhances diagnostic precision.
Report Reading and Clinical Correlation	Reads RCNR manually; extracts procedural/consultation/progression notes mentally.	Microsoft-Phi-2 generates structured clinical notes from unstructured RCNR.	Saves reading and correlation time.	Ensures consistent extraction of clinically relevant text.
Clinical Decision Synthesis	Correlates image findings with clinical notes to formulate a diagnosis and classification.	Bio-BERT interprets RCNR alongside images to assist with classification.	Cognitive load shifted to AI-driven NLP.	Faster, more consistent brain hemorrhage subtype classification.
Hemorrhage Subtype Classification	Manual classification into ICH, EDH, SDH, and IVH.	Multimodal classifier (Tuned-ViT + Bio-BERT) automatically categorizes subtypes.	Eliminates manual subtype labeling.	Enables quicker subtype-specific interventions.
Sentiment and Outcome Reporting	Radiologists may consult with physicians for documentation of prognosis/therapeutic strategy.	Microsoft-Phi-2 generates prognosis and therapeutic sentiment analysis reports automatically.	Reduces multidisciplinary communication overhead.	Timely therapeutic decisions and outcome forecasting.

## Data Availability

The data provided in the manuscript is available from the ROCO-V2 dataset.

## LIST OF ABBREVIATIONS

Bio-BERT: Bio-Bidirectional Encoder Representation Transformer
DL: Deep Learning
EDH: Epidural hemorrhage
ICH: Intracerebral hemorrhage
IH: Intracranial hemorrhage
IVH: Intraventricular hemorrhage
LLM: Large Language Model
M-CLAHE: Modified Contrast Limited Adaptive Histogram Equalization
MJFSA: Multimodal Joint Fusion Sentiment Analysis
POA: Pufferfish Optimization Algorithm
RCNR: Radiology Clinical Narrative Report
ROCO-V2: Radiology Object in Context Version-2
SDH: Subdural Hemorrhage
T-ViT: Tuned-Vision Image Transformer
UMLS: Unified Medical Language Systems

## References

[1] Brain hemorrhage - Symptoms, types, causes, complications, treatment.. https://www.pacehospital.com/brain-hemorrhage-causes-symptoms-types-treatment.

[2] Brain bleed, hemorrhage (intracranial hemorrhage).. https://my.clevelandclinic.org/health/diseases/14480-brain-bleed-hemorrhage-intracranial-hemorrhage.

[3] Hartung M.P., Bickle I.C., Gaillard F., Kanne J.P. (2020). How to create a great radiology report.. Radiographics.

[4] Bruce S.S., Pawar A., Liao V., Merkler A.E., Liberman A.L., Navi B.B., Iadecola C., Kamel H., Zhang C., Murthy S.B. (2025). Nontraumatic intracranial hemorrhage and risk of incident dementia in US medicare beneficiaries.. Stroke.

[5] Kiefer J., Kopp M., Ruettinger T., Heiss R., Wuest W., Amarteifio P., Stroebel A., Uder M., May M.S. (2023). Diagnostic accuracy and performance analysis of a scanner-integrated artificial intelligence model for the detection of intracranial hemorrhages in a traumatology emergency department.. Bioengineering.

[6] Sengupta J., Alzbutas R., Falkowski-Gilski P., Falkowska-Gilska B. (2023). Intracranial hemorrhage detection in 3D computed tomography images using a bi-directional long short-term memory network-based modified genetic algorithm.. Front. Neurosci..

[7] Denecke K., Reichenpfader D. (2023). Sentiment analysis of clinical narratives: A scoping review.. J. Biomed. Inform..

[8] Rajput A. (2020). Natural language processing, sentiment analysis, and clinical analytics.. Innovation in Health Informatics.

[9] Mohamad Beigi O., Moattar M.H. (2021). Automatic construction of domain-specific sentiment lexicon for unsupervised domain adaptation and sentiment classification.. Knowl. Base. Syst..

[10] Unnithan A.K.A., Das J.M., Mehta P. (2023). Hemorrhagic stroke.. StatPearls.

[11] Magid-Bernstein J., Girard R., Polster S., Srinath A., Romanos S., Awad I.A., Sansing L.H. (2022). Cerebral hemorrhage: Pathophysiology, treatment, and future directions.. Circ. Res..

[12] Hemorrhagic stroke: Intracerebral hemorrhage.. https://www.physio-pedia.com/Hemorrhagic_Stroke:_Intracerebral_Hemorrhage.

[13] Raposo N., Zanon Zotin M.C., Seiffge D.J., Li Q., Goeldlin M.B., Charidimou A., Shoamanesh A., Jäger H.R., Cordonnier C., Klijn C.J.M., Smith E.E., Greenberg S.M., Werring D.J., Viswanathan A. (2023). A causal classification system for intracerebral hemorrhage subtypes.. Ann. Neurol..

[14] (2024). Sentiment analysis in healthcare: Transforming patient feedback into actionable insights.. https://www.repugen.com/blog/sentiment-analysis-in-healthcare.

[15] Eberle T.S., Rebitzke Eberle V. (2019). Finding self, sense, and sense making after a cerebral hemorrhage.. J. Appl. Soc. Sci..

[16] Bark D., Basu J., Toumpanakis D., Burwick Nyberg J., Bjerner T., Rostami E., Fällmar D. (2024). Clinical impact of an AI decision support system for detection of intracranial hemorrhage in CT scans.. Neurotrauma Rep..

[17] Babi M.A., Mayberry W., Koriesh A., Nouh A. (2025). Editorial: Neuro-imaging in intracerebral hemorrhage: updates and knowledge gaps.. Front. Neurosci..

[18] Del Gaizo A.J., Osborne T.F., Shahoumian T., Sherrier R. (2024). Deep learning to detect intracranial hemorrhage in a national teleradiology program and the impact on interpretation time.. Radiol. Artif. Intell..

[19] Flanders A.E., Prevedello L.M., Shih G., Halabi S.S., Kalpathy-Cramer J., Ball R., Mongan J.T., Stein A., Kitamura F.C., Lungren M.P., Choudhary G., Cala L., Coelho L., Mogensen M., Morón F., Miller E., Ikuta I., Zohrabian V., McDonnell O., Lincoln C., Shah L., Joyner D., Agarwal A., Lee R.K., Nath J. (2020). RSNA-ASNR 2019 intracranial hemorrhage CT Annotators. Construction of a machine learning dataset through collaboration: the RSNA 2019 brain CT hemorrhage challenge.. Radiol. Artif. Intell..

[20] Song C., Zhao Q., Li J., Yue X., Gao R., Wang Z. HemSeg-200: A Voxel-Annotated Dataset for Intracerebral Hemorrhages Segmentation in Brain CT Scans.. Proceedings of the 2024 IEEE International Conference on Systems, Man, and Cybernetics (SMC).

[21] Denecke K., Deng Y. (2015). Sentiment analysis in medical settings: New opportunities and challenges.. Artif. Intell. Med..

[22] Mitra J., Qiu J., MacDonald M., Venugopal P., Wallace K., Abdou H., Richmond M., Elansary N., Edwards J., Patel N., Morrison J., Marinelli L. (2022). Automatic hemorrhage detection from color doppler ultrasound using a generative adversarial network (GAN)-based anomaly detection method.. IEEE J. Transl. Eng. Health Med..

[23] Wang X., Cai W., Shen D., Huang H. (2018). Temporal correlation structure learning for MCI conversion prediction.. Medical Image Computing and Computer Assisted Intervention – MICCAI 2018.

[24] Saito M., Matsumoto E., Saito S. Temporal generative adversarial nets with singular value clipping.. Proceedings of the IEEE International Conference on Computer Vision.

[25] Sanglerdsinlapachai N., Plangprasopchok A., Ho T.B., Nantajeewarawat E. (2021). Improving sentiment analysis on clinical narratives by exploiting UMLS semantic types.. Artif. Intell. Med..

[26] Ahmad I.S., Dai J., Xie Y., Liang X. (2025). Deep learning models for CT image classification: a comprehensive literature review.. Quant. Imaging Med. Surg..

[27] Rückert J., Bloch L., Brüngel R., Idrissi-Yaghir A., Schäfer H., Schmidt C.S., Koitka S., Pelka O., Abacha A.B., G Seco de Herrera A., Müller H., Horn P.A., Nensa F., Friedrich C.M. (2024). Rocov2: Radiology objects in context version 2, an updated multimodal image dataset.. Sci. Data.

[28] Siniscalchi A., Gray C., Malferrari G. (2021). Ultrasound diagnostic method in vascular dementia: Current concepts.. Curr. Med. Imaging.

[29] Manouvelou S., Koutoulidis V., Tsougos I., Tolia M., Kyrgias G., Anyfantakis G., Moulopoulos L.A., Gouliamos A., Papageorgiou S. (2020). Differential diagnosis of behavioral variant and semantic variant of frontotemporal dementia using visual rating scales.. Curr. Med. Imaging.

[30] Prakash D., Madusanka N., Bhattacharjee S., Kim C.H., Park H.G., Choi H.K. (2021). Diagnosing Alzheimer’s disease based on multiclass MRI scans using transfer learning techniques.. Curr. Med. Imaging.

[31] Cui J., Bian W., Wang J., Niu J. (2024). Advances in imaging techniques of the blood-brain barrier and clinical application.. Curr. Med. Imaging Rev..

[32] Phan T.C., Phan A.C. (2024). Automatic detection and segmentation of intracranial hemorrhage based on improved U-Net model.. Curr. Med. Imaging.

[33] Kumaravel P., Mohan S., Arivudaiyanambi J., Shajil N., Venkatakrishnan H.N. (2021). A simplified framework for the detection of intracranial hemorrhage in CT brain images using deep learning.. Curr. Med. Imaging.

[34] Kaliannan S., Rengaraj A. (2024). Differentiating the presence of brain stroke types in MR images using CNN architecture.. Curr. Med. Imaging.

[35] Yin X., Ciren D., Guojie C., Zhang G., Wang J., Zhang H. (2025). Intracranial structural malformations in children in tibet: CT and MRI findings in a single tertiary center.. Curr. Med. Imaging.

